# Assessment of organochlorine pesticide residues in soils and drinking water sources from cocoa farms in Ghana

**DOI:** 10.1186/s40064-016-2352-9

**Published:** 2016-06-24

**Authors:** Benedicta Y. Fosu-Mensah, Elvis D. Okoffo, Godfred Darko, Christopher Gordon

**Affiliations:** Institute for Environment and Sanitation Studies (IESS), University of Ghana, P. O. Box 209, Legon, Accra, Ghana; Department of Chemistry, Kwame Nkrumah University of Science and Technology, Kumasi, Ghana

**Keywords:** Cocoa, Organochlorine, Soil, Drinking water, Pesticides residue

## Abstract

Residues of organochlorine pesticides were determined in soils and drinking water sources in cocoa growing areas in Ghana. Soil samples analysed showed the presence of four organochlorine pesticide residues namely lindane (0.005–0.05 mg/kg), beta-HCH (<0.01–0.05 mg/kg), dieldrin (0.005–0.02 mg/kg), and *p,p*′-DDT (0.005–0.04 mg/kg), with dieldrin occurring most frequently. Similarly, organochlorine pesticide residues detected in the water samples were lindane (0.01–0.03 µg/l), alpha-endosulfan (0.01–0.03 µg/l), endosulfan-sulphate (0.01–0.04 µg/l), dieldrin (0.01–0.03 µg/l) and *p,p*′-DDT (0.01–0.04 µg/l), with heptachlor occurring most frequently. The concentrations of the detected organochlorine residues in the soil samples were below their respective US maximum residues limits (MRLs) for agricultural soils, except for lindane recorded at Kwakuanya (S4) and beta-HCH recorded at Krakrom (S3) and Kwakuanya (S4). Similarly, the organochlorine pesticide residues recorded in the water samples were below and within their respective WHO MRLs for drinking water except for alpha-endosulfan at Diabaa (S2) and Kwakuanya (S4) at distance 0–15 m and Kwakuanya (S4) at distance 16–30 m, endosulfan-sulfate at Nkrankwanta (S1) and Diabaa (S2) at distance 0–15 m and heptachlor at Krakrom (S3) at distance 16–30 m which were above their WHO MRLs. The presence of the banned organochlorine pesticide residues in soil and water samples from the study area indicates that these chemicals are still being used, illegally, on some cocoa farms. Routine monitoring of pesticide residues in the study area is necessary for the prevention, control and reduction of environmental pollution to minimize health risks.

## Background

Cocoa (*Theobroma cacao*), which is used mainly in the production of chocolate, is an important agricultural export commodity in Ghana (Ntiamoah and Afrane 2008). The country is currently ranked second in the world, after her western neighbour Cote d’Ivoire in cocoa production (Anang et al. 2013). Cocoa serves as the major source of revenue for the provision of socio-economic infrastructure in the country. In terms of employment, the industry employs about 60 % of the national agricultural labour force and contributes about 70–100 % of their annual household incomes (Appiah 2004; Anim-Kwapong and Frimpong 2004; Ntiamoah and Afrane 2008; Afrane and Ntiamoah 2011). However, insect pests and diseases pose a major challenge to the production of the crop resulting in adverse consequences on the country’s economy. In an attempt to reduce the incidence of insect pests and diseases, a large number of pesticides including organochlorines are usually applied on farms.

Organochlorine pesticides have long residual action and persist in the environment for long periods without losing their toxicity (Agbeve et al. 2014). Many organochlorine pesticides and their metabolites are highly toxic and have been implicated in a wide range of adverse health effects such as cancer, neurological damage, reproductive system deformities, birth defect, and damage to the immune system (Ahlborg et al. 1995; Sosan et al. 2008; Leena et al. 2012). Their environmental concern is further heightened by the fact that they are not easily broken down in the environment, as they resist degradation by chemical, physical, microbiological, and biological means (Darko and Acquaah 2007; Darko et al. 2008). They therefore have the potential to cause damage to ecosystems (Botwe et al. 2012; Agbeve et al. 2014). Organochlorine pesticides are volatile and can move off-site via air-drift and surface runoff to contaminate remote areas (including water bodies) where they have not been used (Miglioranzaa et al. 1999; Kuranchie-Mensah et al. 2012). As a result of their lipophilicity and persistence, organochlorine pesticides bio-accumulate along the food chain posing health threats to human beings (Mbakaya et al. 1994; Manirakiza et al. 2002; Botwe et al. 2012; Kuranchie-Mensah et al. 2012).

The adverse consequences associated with the use of organochlorine pesticides are the reasons why many of them were banned or have their use restricted (Botwe et al. 2012; Asiedu 2013). However, there are evidence of their continuous usage in some developing countries, such as Ghana, due to their efficacy and low cost and possibly due to weak import control and lack of logistics to monitor pesticides (Darko and Acquaah 2007; Asiedu 2013). In the past few years, a number of studies have shown the presence of organochlorine pesticide residues in fish (Darko et al. 2008) water and sediments (Ntow 2001; Darko et al. 2008; Kuranchie-Mensah et al. 2012), fruits and vegetables (Bempah and Donkor 2011; Bempah et al. 2011), meat (Darko and Acquaah 2007) and human fluids (blood and breast milk) (Ntow 2001; William et al. 2008; Tutu et al. 2011) in Ghana.

The Dormaa West District located in the Brong Ahafo Region of Ghana is known to be one of the major cocoa producing regions in Ghana. Over the years, there has been a decline in cocoa yield in the district as a result of insect pests and diseases. In order to increase cocoa productivity in the district, there has been an increased use of pesticides to control insect pests and diseases. However, the regular application and indiscriminate use of these chemicals may pollute the environment (soils and drinking water sources) and affect human health. Unfortunately, there is little information and studies on levels of pesticide residues in soils and drinking water sources within and around cocoa growing farms in the district. This study therefore assessed 15 organochlorine pesticide residues in soils and drinking water sources within and around cocoa farms in the Dormaa West District of the Brong Ahafo Region of Ghana.

## Methods

### Study area and sampling design

**Fig. 1:**
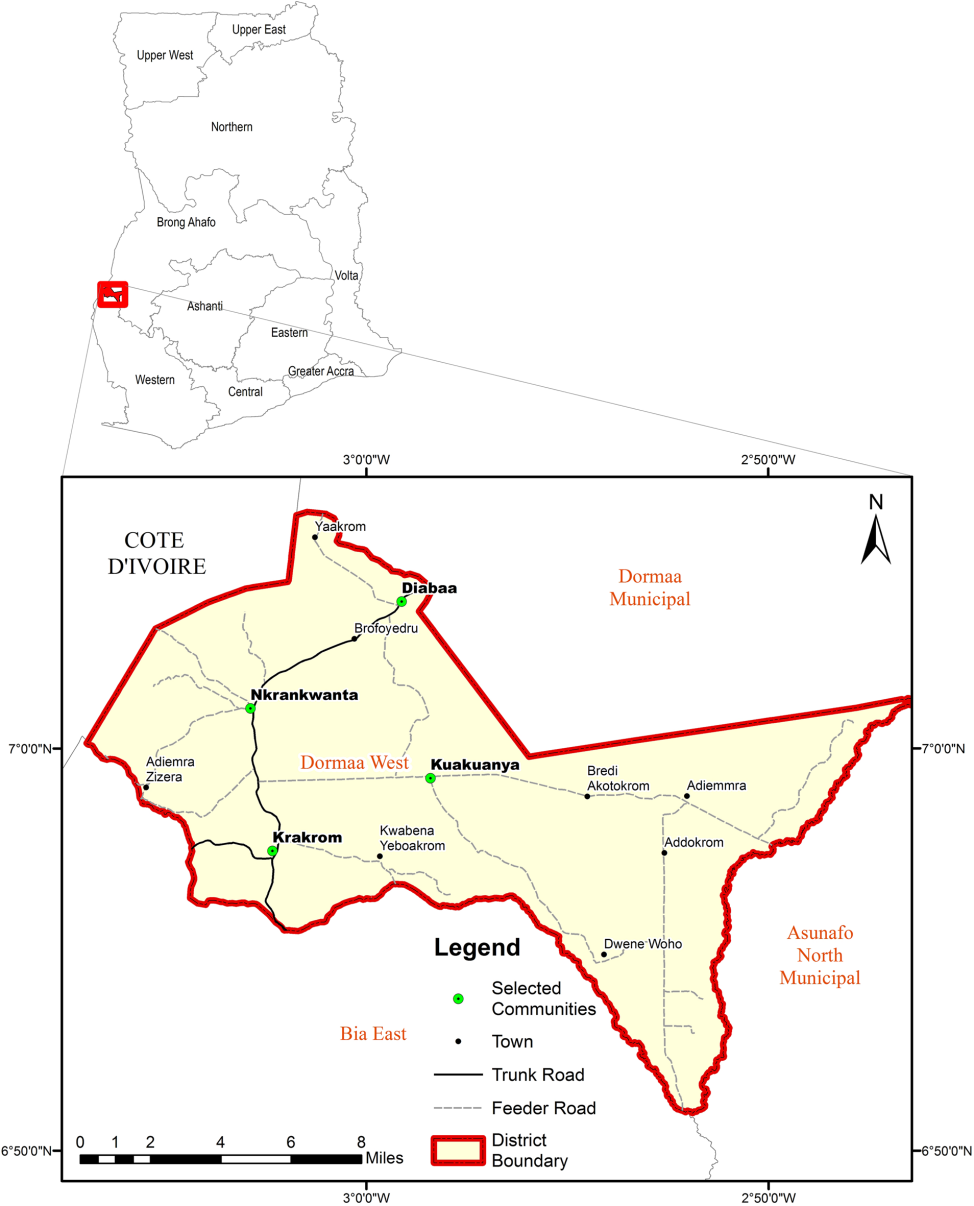
Map of Dormaa West District showing selected communities

The study was conducted in the Dormaa west district located at the western part of the Brong-Ahafo region of Ghana (Fig. 1). It is one of the major cocoa producing districts in the country. The district has a tropical climate with distinct wet and dry season. The dry season spans from November to February and the rainy season from May to September. Mean annual rainfall is between 125 and 175 cm with a mean annual temperature of about 28.1 °C (Ghana Statistical Service 2014).

Four cocoa growing communities were randomly selected from the study area. The communities selected were Nkrankwanta (S1), Diabaa (S2), Krakrom (S3) and Kwakuanya (S4). In each community, four cocoa farms were identified purposively, making a total of 16 sites for the study. The criteria for the selection was based on (1) distance of farm to the nearest drinking water source (hand dug wells) and (2) age of cocoa farm (farms not less than 8 years and not more than 20 years with a history of at least 5 years of pesticides application). Soil and water samples were taken from December, 2014 to February, 2015.

### Collection of soil samples and physicochemical analysis

In each selected cocoa farm, two quadrates of 80 × 80 m were marked. In each quadrate, 5 core soil samples were collected randomly at a depth of 0–20 cm using a soil auger and bulked together to form a composite sample. They were then placed in well-labelled clean plastic polythene bags and transported to the laboratory for analysis. Two soil replicates were collected from each cocoa farm. These gave a total of 32 soil samples for the study area. The samples were air-dried for 5 days and then oven-dried at 105 °C until constant weight was obtained. They were then ground and passed through a 2 mm sieve and sub-samples taken for physicochemical and pesticide residues analysis.

Soil physicochemical properties measured were pH, electrical conductivity, soil particle size distribution, organic carbon/organic matter, total nitrogen, ammonium, nitrate, available phosphorus, available potassium and exchangeable potassium. Soil pH and electrical conductivity were determined in a 1:1 soil to distilled water ratio (McKeague 1978; McLean 1982) using microprocessor pH Meter (Van London Phoenix Electrodes, USA). Particle size analysis of the soil was carried out using Bouyoucos hydrometer (POBEL, Spain) method modified by Day (1965). Soil organic carbon (SOC) was determined by a modified procedure by Walkley and Black (1934). The amount of soil organic matter (SOM) was found by multiplying the percentage C by the factor 1.724 (Walkley and Black 1934). Total soil nitrogen was determined using the micro distillation (Kjeldahl 1883) and titration method (Bremner and Mulvaney 1982). Available potassium was determined using the ammonium acetate method (Feech 1965). The available phosphorus was determined using the Bray 1 procedure (Bray and Kurtz 1945) and exchangeable potassium determined using the flame photometer method (Chapman 1965).

### Collection of water samples

Water samples were collected from hand dug wells located within and around the selected cocoa farms. Wells were selected based on distance to cocoa farms in the order; 0–15 m and 16–30 m whiles wells situated above 30 m were used as the control. These distances were chosen based on the World Health Organization (WHO) recommendation that hand dug wells should be at least 30 m (100 ft) away from an agricultural field where chemicals (pesticides and fertilizers) are handled (Harris et al. 1996; World Health Organization, WHO 2004). Water samples were collected from two hand-dug wells for each of the distance categories (between 0–15 and 16–30 m) within each selected community. Water samples were also collected from three (3) open wells (above 30 m from farms) as control. Three replicates were collected from each well making a total of sixty-four (64) water samples. A water sampler was used to collect water samples into 1.5 l and 500 ml pre-cleaned polyethylene sample bottles for pesticide residues and physicochemical analysis respectively.

### Determination of pesticides residue in soil and water samples

#### Chemicals and reagents used

Soil and water samples were analysed for 15 organochlorine pesticides (OCPs) (β-HCH, γ-HCH, δ-HCH, heptachlor, aldrin, γ-chlordane, *p,p′*-DDE, *p,p′*-DDT, *p,p′*-DDD, dieldrin, endrin, α-endosulfan, β-endosulfan, endosulfan-sulfate and methoxychlor). The individual OCPs certified reference standards with certified purity of 99 % were purchased from Dr. Ehrenstorfer GmbH (Augsburg, Germany). Pesticides grade acetonitrile and dichloromethane were purchased from BDH, England. Silica gel adsorbents were obtained from Phenomenex, USA. All other reagents and solvents used were of analytical grade purchased from BDH, England and included: anhydrous magnesium sulphate, anhydrous sodium sulphate, acetone, ethyl acetate, and saturated sodium chloride.

#### Extraction of soil samples

The extraction of the soil samples was carried out by the method described by Frimpong et al. (2013), with slight modification from the Ghana Standard Authority (GSA) Pesticide Residues Laboratory Protocols. Ten grams (10 g) of the sub-soil samples were weighed and transferred into 250 ml separating flasks. A 10 ml of acetonitrile was added and the corked flasks sonicated (Grant XUB 18UK) for 5 min. An additional 10 ml of acetonitrile was added, and the separating flasks closed tightly. The content of the flasks were placed on a horizontal mechanical shaker (Ika-Werke HS 501 Digital), and was set to shake continuously for 30 min at 300 mot/min, and allowed to stand for 10 min to sufficiently separate the phases or layers. The supernatants (organic layers) were carefully transferred into 50 ml centrifuge tubes for centrifugation (Thermo/CR3i Multifunction) at 3000 rpm for 5 min. A 10 ml aliquot of the supernatants (organic phases/top layers) equivalent to 5.0 g soil weight were pipetted and dried/passed over 5 g anhydrous sodium sulphates through a filter paper into 50 ml round-bottom flasks. Then, 5 ml of acetonitrile was used to rinse the salt into the round-bottom flasks. The concentrates were then adjusted to about 2 ml using the rotary film evaporator (Buchi Ratovapor R-210, USA) at 35 °C, and made ready for the clean-up step.

#### Extraction of water samples

The extraction technique employed for the water samples in this work was as described by Afful et al. (2013) and Gbeddy et al. (2015), with slight modification from the Ghana Standard Authority (GSA) Pesticide Residues Laboratory protocols. pH of the water samples was checked to ensure that it was neutral to avert the organochlorine pesticides from undergoing chemical reactions. After filtration of water samples through 0.45 ml fiber glass filters (WHATMAN) to remove debris and suspended material, 1000 ml portion of the filtered water sample were transferred into a 2 l capacity glass-separating flasks. A 30 ml of saturated sodium chloride solution (NaCl) was added to produce a salt out effect. The samples were then thoroughly mixed by inverting the flask 3–4 times. A 100 ml of dichloromethane as extraction solvent was added to each sample and vigorously shaken manually for 2–3 min, while releasing the pressure intermittently. The phases were allowed to separate for 5 min and the dichloromethane extracts (organic layers) were separated from the aqueous layers. The extraction for each water sample was repeated two times with 100 ml of dichloromethane and the organic layers put together, and dried over anhydrous magnesium sulphate through filter papers into 50 ml round bottom flasks. The extracts from the water samples were then concentrated on rotary film evaporator (Buchi Ratovapor R-210, USA) to about 2 ml prior to clean up.

#### Clean up of soil extracts

Silica (1000 mg/6 ml) cartridges which have 1 g layer of anhydrous magnesium sulphate weighed on top was conditioned using 6 ml acetonitrile. A 50 ml pear shape flasks were placed under the columns in a vacuum manifold, and the concentrated extracts loaded onto the cartridges. The extracts were allowed to filter and the cartridges eluted with 10 ml of acetonitrile with slight intermittent vacuum use. The eluents collected were then concentrated to dryness using the rotary film evaporator (Buchi Ratovapor R-210) set at 40 °C. The extracts were re-dissolved in 1 ml ethyl acetate by pipetting and the dissolved extracts carefully transferred into labelled 2 ml chromatography (GC) standard opening vials prior to quantitation by gas chromatography (GC) (Varian Association Inc. USA) equipped with Electron Capture Detector (ECD). All extracts were kept frozen until quantification was achieved.

#### Clean up of water extracts

Silica (1000 mg/6 ml) cartridges which have 2 g layer of anhydrous sodium sulphate weighed on top was conditioned with 6 ml dichloromethane. The concentrated extracts were then loaded onto the cartridges, and 100 ml round-bottom flasks were placed under the columns to collect the eluates. A 20 ml dichloromethane was then used to elute the cartridges afterwards. The eluents collected were concentrated to dryness using the rotary film evaporator (Buchi Ratovapor R-210) set at 40 °C. The extracts were re-dissolved in 1 ml ethyl acetate by pipetting and carefully transferred into 2 ml standard opening vials prior to quantitation by gas chromatography (GC) (Varian Association Inc. USA) equipped with Electron Capture Detector (ECD). All extracts were kept frozen until quantification was achieved.

#### Determination of organochlorine pesticides residues

The final extracts were analysed using a Varian CP-3800 gas chromatograph (Varian Association Inc. USA) equipped with combiPAL Autosampler and ^63^Ni electron capture detector. The GC conditions used for the analysis were capillary column coated with VF-5 (30 m + 10 m EZ guard column × 0.25 mm internal diameter, 0.25 µm film thickness). The injector and detector temperature were set at 270 and 300 °C respectively. The oven temperature was programmed as follows: 70 °C held for 2 min, ramp at 25 °C/min to 180 °C, held for 1 min, and finally ramp at 5 °C/min to 300 °C. The GC conditions and the detector response were adjusted so as to match the relative retention times and response as spelt out by the Japanese analytical methods for agricultural chemicals (Syoku-An 2006). Nitrogen was used as carrier gas at a flow rate of 1.0 ml/min and detector make-up gas of 29 ml/min. The injection volume of the GC was 1.0 μl. The total run time for a sample was 31.4 min.

#### Quality assurance and quality control

All glassware used for analysis were rigorously washed with detergent, rinsed with distilled water and thoroughly rinsed with analytical grade acetone and dried overnight in an oven at 150 °C. The glassware were then removed and allowed to cool down and stored in dust-free cabinets.

The quality of organochlorine pesticide residues was guaranteed through the analysis of solvent blanks, procedural matrix blanks and duplicate samples. All reagents used during the analysis were exposed to same extraction procedures and solvents used were run to verify for any interfering substances within the runtime. For the solvent blank in each extraction procedure, no values were obtained for the analytes of interest. Recalibration curves were run with each batch of samples to check that the regression coefficient was kept around r^2^ = 0.990. A fortification levels of 0.005 mg/kg and 0.01 μg/l of standards mixture were chosen before the analysis to evaluate the recovery of the compounds in the soil and water samples analysed respectively. Fortified samples were determined with good recoveries. The efficiency of the analytical methods (the extraction and clean-up methods) was determined by recoveries of an internal standard. The mean recoveries of internal standards ranged between 70 and 119 % for all the compounds analysed in the soil and water samples. These recovery values shows that the method used is reproducible.

The residue levels of organochlorine pesticides were quantitatively determined by the external standard method using their peak areas. Measurement was carried out within the linear range of the detector. The peak areas whose retention times coincided with the standards were extrapolated on their corresponding calibration curves to obtain the concentration. The limit of detection of the pesticide residues determined was based on the extract of the fortified samples. One out of each concentration that gave a response three times the standard deviation of the least fortified sample was used to estimate the statistical significance of differences between low level analyte responses and the combined uncertainties in both the analyte and the background measurement. The limits of detection (LOD) for the organochlorine pesticide residues detected in the soil and water samples were 0.005 mg/kg and 0.01 μg/l, respectively.

### Data analysis

Statistical Package for Social Sciences (SPSS) software version 20.0 was used to generate the means of the results. One-way Analysis of variance (ANOVA) was used to test for the significant differences and similarities between the physicochemical properties, and the concentrations of pesticide residues. Significant means obtained were separated by least significant difference at 5 % significance level. A Pearson correlation analysis was carried out to establish the correlation between the physicochemical parameters of soil and the concentrations of pesticides detected. The statistical significance tests were carried at 5 % confidence level (*p* < 0.05).

## Results and discussion

### Physicochemical properties of soil samples from the study area

Table 1 presents the summary of the physicochemical properties of soil samples from the study sites. Soil pH is one of the factors which influences the bio-availability and transport of pesticides in soils (Aiyesanmi et al. 2008). The measured pH of the soils ranged from 7.56 at Nkrankwanta (S1) to 8.49 at Krakrom (S3) with a mean value of 8.07 ± 0.33, indicating that the soils were generally alkaline. This may be as a result of high concentrations of sodium and calcium in the soils (Aikpokpodion 2010). There were significant differences (*p* < 0.05) in mean pH of soil among the various sampled sites. However, when the least significant difference (LSD) was used to compare the means, there were no differences in pH between Nkrankwanta (S1) and Diabaa (S2), and Krakrom (S3) and Kwakuanya (S4).

**Table 1:** Summary of soil physicochemical properties

Sites	Nkrankwanta (1)		Diabaa (2)		Krakrom (3)		Kwakuanya (4)		Total mean
Parameters	Range	Mean ± SD	Range	Mean ± SD	Range	Mean ± SD	Range	Mean ± SD	Mean ± SD
pH	7.34–7.80	7.56 ± 0.23	7.73–7.96	7.88 ± 0.13	8.46–8.52	8.49 ± 0.03	8.21–8.39	8.34 ± 0.04	8.07 ± 0.33
Electrical conductivity (µS/cm)	138.0–296.0	210.0 ± 79.8	163.0–282.0	242.0 ± 68.4	183.0–239.0	203.0 ± 30.9	156–283	204.0 ± 68.9	214.8 ± 14.3
Organic carbon (%)	0.89–1.95	1.38 ± 0.53	5.67–5.99	5.83 ± 0.16	5.78–6.14	5.94 ± 0.18	5.78–6.46	6.12 ± 0.35	4.83 ± 1.78
Organic matter (%)	1.54–3.36	2.38 ± 0.92	9.78–10.3	10.0 ± 0.28	9.96–10.6	10.3 ± 0.31	9.96–11.1	10.6 ± 0.60	8.32 ± 3.07
Available potassium (mg/kg)	0.39–0.95	0.59 ± 0.30	0.45–0.50	0.48 ± 0.03	0.36–0.89	0.64 ± 0.27	0.21–0.50	0.35 ± 0.15	0.52 ± 0.10
Total nitrogen (%)	1.61–2.63	2.10 ± 0.51	1.74–2.46	2.13 ± 0.36	1.57–2.10	1.87 ± 0.27	1.51–1.79	1.64 ± 0.14	1.94 ± 0.18
Phosphorus (mg/kg)	0.55–0.70	0.64 ± 0.08	0.50–0.91	0.71 ± 0.21	0.55–0.69	0.63 ± 0.07	2.40–2.50	2.47 ± 0.06	1.11 ± 0.70
NH_4_^+^ (mg/l)	23.1–46.9	38.9 ± 13.7	30.1–42.0	34.8 ± 6.35	43.4–48.3	45.0 ± 2.83	35.0–49.0	42.9 ± 7.18	40.4 ± 3.49
NO_3_^−^ (mg/l)	23.8–29.4	25.7 ± 3.23	33.6–45.5	40.1 ± 6.04	38.5–44.1	40.6 ± 3.05	28.0–30.1	28.9 ± 1.07	33.8 ± 5.93
Exchangeable potassium (cmol/kg)	0.20–0.26	0.24 ± 0.03	0.27–0.51	0.43 ± 0.14	0.38–0.51	0.60 ± 0.27	0.21–1.15	0.67 ± 0.47	0.49 ± 0.15
Sand (%)	50.1–83.3	65.9 ± 16.7	59.7–80.5	67.8 ± 11.0	59.5–60.4	59.8 ± 0.47	57.0–69.0	63.3 ± 6.04	64.2 ± 2.68
Clay (%)	12.5–35.0	24.2 ± 11.3	7.50–15.0	11.7 ± 3.81	12.5–17.5	15.8 ± 2.89	12.5–12.5	12.5 ± 0.00	16.1 ± 4.43
Silt (%)	4.20–14.9	9.96 ± 5.41	12.0–25.3	20.5 ± 7.35	22.1–28.0	24.3 ± 3.25	18.5–30.5	24.2 ± 6.04	19.7 ± 5.23
Texture		Sandy-clay-loam		Sandy-loam		Sandy-loam		Sandy-loam	

*SD* standard deviation

The measured conductivity ranged from 203.0 to 242.0 µS/cm with a mean value of 214.8 ± 14.3 µS/cm. There was however no significant difference (*p* > 0.05) in mean conductivity of soil among the various sampled sites.

Despite the high rate of decomposition of organic matter often found in tropical soils, soils from the study sites were characterized by high organic carbon (OC) and organic matter (OM) content perhaps due to the high influx of litter and microbial activities (Dankyi et al. 2014). Soil organic carbon and organic matter are known to influence the dynamics and behaviour of both inorganic and organic pollutants in soils (Gale et al. 2004; Aiyesanmi et al. 2008). Percent OC ranged from 1.38 % at Nkrankwanta (S1) to 6.12 % at Kwakuanya (S4) with a mean value of 4.83 % ± 1.78. There were significant differences (*p* < 0.05) in mean percent organic carbon among the sampled sites. However, the LSD revealed significant difference in percent organic carbon among the following sampled sites; Diabaa (S2), Krakrom (S3), Kwakuanya (S4) and Nkrankwanta (S1).

Similarly, percent OM ranged from 2.38 % at Nkrankwanta (S1) to 10.6 % at Kwakuanaya (S4) with a mean value of 8.32 % ± 3.07. Percent organic matter of the soil differed significantly (*p* < 0.05) among the various sampled sites. However, when LSD was used to compare the means, there were differences in percent organic matter among the following sampled sites; S2, S3, S4 and S1.

The mean of the available potassium concentrations was found to be 0.52 ± 0.10 mg/kg. There were no significant differences (*p* > 0.05) in mean potassium among the various sampled sites. The measured percentage nitrogen content of the soils ranged from 1.64 % at Kwakuanya to 2.13 % at Diabaa with a mean of 1.94 % ± 0.18. There were however, no significant differences (*p* > 0.05) in mean percent nitrogen among the various sampled sites. The available phosphorus values ranged from 0.63 mg/kg at S3 to 2.47 mg/kg at S4 with a mean value of 1.11 ± 0.70 mg/kg. There were significant (*p* < 0.05) sites difference for available phosphorus. Analysis of variance (ANOVA) showed there were differences in available phosphorus among S2, S3, S1 and S4.

The measured ammonium concentration ranged from 34.8 mg/l at S2 to 45.0 mg/l at S3 with a mean of 40.4 ± 3.49 mg/l. There were no significant differences in mean ammonium concentration (*p* > 0.05) among the various sampled sites. Analysis of variance at 95 % confidence interval revealed significant difference (*p* < 0.05) in mean soil nitrate among the various sites. The measured nitrate ranged from 25.7 mg/l at Nkrankwanta to 40.6 mg/l at Krakrom with a mean of 33.8 ± 5.93 mg/l. Least significant difference showed there was no difference in nitrate between Kwakuanya and Nkrankwanta. The exchangeable K of the soil samples ranged from 0.24 cmol/kg at Nkrankwanta to 0.67 cmol/kg at Kwakuanya with a mean value of 0.49 ± 0.15 cmol/kg. Statistically, there were no significant differences in mean exchangeable potassium (*p* > 0.05) among the sites.

In general, the content of sand was greater than 50 % at all the sampled sites and varied widely. However, there was no significant sites difference (*p* > 0.05) for percent sand. Also, percent clay was lower than 20 % in more than half of the analysed soil samples and there were statistically significant sites difference (*p* < 0.05). However, the comparison of the means, showed no significant difference in percent clay among Kwakuanya, Nkrankwanta, Diabaa, and Krakrom. There were however significant sites difference in mean percent silt (*p* < 0.05). Silt content of the soil was greater than 20 % at all the sampled sites except S1 which recorded a mean value of 9.96 %. When the LSD was used to compare the means, there were no significant difference in percent silt between S2 and S3, but was statistically different from S1. The soil particle size distribution reveals the textural class of the soils as mainly sandy loam and sandy-clay-loam according to the United States Department of Agriculture classification system (USDA 1987).

### Organochlorine pesticide residues in soil samples

Table 2 presents the concentrations of organochlorine pesticides measured in the soils samples. Soil samples analysed showed the presence of four (4) out of the fifteen (15) organochlorine pesticides namely lindane, beta-HCH, dieldrin, and *p,p′*-DDT. There were however no statistically significant sites difference (*p* > 0.05) in mean concentrations of the detected residues in the soil samples.

**Table 2:** Result of detected organochlorine pesticide residues in soil samples

Sites	Nkrankwanta (S1)		Diabaa (S2)		Krakrom (S3)		Kwakuanya (S4)		Total mean	US MRLs
Pesticides (mg kg^−1^)	Range	Mean ± SD	Range	Mean ± SD	Range	Mean ± SD	Range	Mean ± SD	Mean ± SD	
Lindane	ND–0.03	0.03 ± 0.00	ND	ND	ND–0.04	0.04 ± 0.00	ND–0.05	0.05 ± 0.00	0.04 ± 0.01	0.04 (Max)
Dieldrin	ND	ND	0.02–0.03	0.02 ± 0.00	ND–0.02	0.02 ± 0.00	0.01–0.03	0.02 ± 0.01	0.02 ± 0.00	0.02 (Max)
Beta-HCH	ND–< 0.01	<0.01	ND–0.03	0.03 ± 0.00	ND–0.05	0.05 ± 0.00	ND–0.04	0.04 ± 0.00	0.03 ± 0.01	0.03 (Max)
*p*,*p*′-DDT	ND–0.02	0.04 ± 0.02	ND–0.02	0.02 ± 0.00	ND	ND	ND–0.03	0.03 ± 0.00	0.03 ± 0.01	0.05 (Max)

*SD* standard deviation, *ND* not-detected, limit of detection = 0.005 mg/kg, *US MRLs* United States maximum residue limits for pesticides in agricultural soils

Lindane was detected in 31.3 % of the soil samples analysed at a mean concentration of 0.04 ± 0.01 mg/kg. However, with the exception of Nkrankwanta (S1) which recorded a mean lindane concentration of 0.03 mg/kg, Krakrom (S3) and Kwakuanya (S4) recorded mean concentrations of 0.04 and 0.05 mg/kg which were comparable to and higher than the US MRLs of 0.04 mg/kg for agricultural soils, respectively. The presence of lindane in the soil samples may suggest the historical use or illegal use of technical HCH mixtures in the study area, since technical lindane have been officially discontinued as restricted chemical for use on cocoa since 2002 (Adu-kumi et al. 2010; Afful et al. 2010). The mean lindane value recorded in this study was higher than the mean values of 0.002 and 0.0001 mg/kg recorded in soil samples from cocoa farms in Kade in the Eastern Region of Ghana (Agyen 2011) and Ilawe-Ekiti, Ekiti State, Nigeria (Olayinka 2013), respectively. On the contrary, the mean concentration reported in this study was lower than the 8.60 and 0.257 mg/kg reported by Bentum et al. (2006) and Aiyesanmi and Idowu (2012) in soils from selected cocoa farms in the Central Region of Ghana and Ondo State of Nigeria, respectively.

The isomer of benzene hexachloride or hexa-chlorocyclohexane (HCH), beta-HCH occurred in 26.6 % of the soil samples analysed, with concentrations ranging from <0.01 mg/kg at Nkrankwanta to 0.05 mg/kg at Krakrom. The mean concentration (0.03 ± 0.01 mg/kg) obtained was comparable to the US MRL of 0.03 mg/kg for agricultural soils. However, the mean beta-HCH concentrations observed at Krakrom (0.05 mg/kg) and Kwakuanya (0.04 mg/kg) were above the MRL of 0.03 mg/kg for agricultural soils whiles soils from Diabaa and Nkrankwanta recorded mean concentration of 0.03 mg/kg and <0.01 mg/kg which were comparable to and below the US MRL for agricultural soils, respectively. The measured concentrations of beta-HCH in the studied soils confirmed the previous use or current use of lindane in the study area, since it is the only BHC or HCH isomer with insecticidal action (Aiyesanmi and Idowu 2012). The mean residue of beta-HCH recorded in this study was higher than the mean value of 0.001 mg/kg reported in soil samples from selected cocoa farms in Kade in the Eastern Region of Ghana (Agyen 2011), but lower than the mean value of 0.617 mg/kg reported in soils from selected cocoa farms in Ondo State, Nigeria (Aiyesanmi and Idowu 2012).

Dieldrin occurred in 62.5 % of the soil samples analysed from the study area. The mean concentration of dieldrin was 0.02 ± 0.00 mg/kg and ranged from below detection at Nkrankwanta to a maximum of 0.02 mg/kg at Diabaa, Krakrom and Kwakuanya. The concentrations of dieldrin recorded were all comparable to the US MRL of 0.02 mg/kg for agricultural soils. Dieldrin is an epoxide of aldrin. It may also form as a biological metabolite of aldrin in soils (Miles et al. 2009; Hogarh et al. 2014). The detection of dieldrin in the soil samples suggests a higher rate of conversion or decomposition of aldrin to dieldrin in the environment, as aldrin was not detected in the soil samples. In addition, the presence of dieldrin could be attributed to the current or previous use of dieldrin as insecticide. The mean concentration of dieldrin recorded in this study was relatively lower than the mean value of 0.197 mg/kg reported in soils from selected cocoa farms in Ondo State Central District, Nigeria (Aiyesanmi and Idowu 2012). On the contrary, the mean concentration of dieldrin recorded in this study was higher than the mean of 0.0032 mg/kg reported in soils from selected cocoa farms in Kade in the Eastern Region of Ghana (Agyen 2011).

*p,p′*-DDT occurred in 50 % of the soil samples analysed at a mean concentration of 0.03 ± 0.01 mg/kg, which was lower than the US MRL of 0.05 mg/kg for agricultural soils. The presence of *p,p′*-DDT in the soil samples might be as a result of its previous use in the studied farms. However, the fact that *p,p′*-DDT residue occurred in 50 % of the studied soils suggests that most cocoa farmers in the study area still use technical DDT even after it has been officially discontinued as restricted chemical for use on cocoa since 1985 (Adu-kumi et al. 2010). According GNIP (2007), DDT has officially not been imported into Ghana since year 2002, and as such, any incident of current use may be attributed to illegal importation. The continued use of DDT by the farmers could be due to addiction to the insecticide based on their easy accessibility and efficacy. It could also be that, new pesticides having DDT as its active ingredient but with different brand names were sold to farmers. The mean concentration of *p,p′*-DDT reported in this study was higher than the mean values of 0.003 and 0.007 mg/kg reported in soils from Kade in Ghana (Agyen 2011) and from Ekiti State in Nigeria (Olayinka 2013), respectively.

### Organochlorine pesticide residues in water samples

The organochlorine pesticide residues detected in the water samples were lindane, α-endosulfan, endosulfan-sulphate, dieldrin and *p,p′*-DDT (Table 3). Similarly, there were no statistically significant differences (*p* > 0.05) in mean values of the pesticide residues detected in relation to distance of water sources to cocoa farms.

**Table 3:** Results of detected organochlorine pesticide residues in drinking water samples with respect to distances to cocoa farm

Distance	0–15 m		16–30 m		Above 30 m		Total mean	WHO MRLs
Pesticides (µg l^−1^ **)**	Range	Mean ± SD	Range	Mean ± SD	Range	Mean ± SD	Mean ± SD	
Lindane	ND	ND	ND–0.04	0.03 ± 0.01	ND	ND	0.03 ± 0.00	2.00 (Max)
α-Endosulfan	ND–0.03	0.03 ± 0.01	ND–0.03	0.02 ± 0.01	ND	ND	0.03 ± 0.00	0.01 (Max)
Dieldrin	ND–0.03	0.03 ± 0.00	ND	ND	ND	ND	0.03 ± 0.00	0.03 (Max)
p,p*′*-DDT	ND–0.03	0.03 ± −0.00	ND–0.05	0.04 ± 0.01	ND	ND	0.04 ± 0.01	2.00 (Max)
Endosulfan-sulfate	ND–0.04	0.03 ± 0.01	ND	ND	ND	ND	0.03 ± 0.01	0.01 (Max)
Heptachlor	ND–0.03	0.02 ± 0.01	ND–0.04	0.02 ± 0.01	< 0.01–< 0.01	<0.01	0.02 ± 0.00	0.03 (Max)

*SD* standard deviation and *ND* non-detected, Limit of detection = 0.01 µg/L, *WHO MRLs* World Health Organization maximum residue limit for pesticides in water

Lindane was not detected in the water samples analysed from distances 0–15 m and above 30 m (control). However, it was detected in 3 out of the 4 sampled sites within distances 16–30 m from cocoa farms with a mean value of 0.03 mg/kg, which was below the WHO permissible limit of 2.00 µg/l for drinking water. In addition, the mean concentrations recorded at the three sites were all below the WHO permissible limit of 2.00 µg/l for drinking water. The presence of lindane in the water samples confirmed the previous use or continuous illegal use of the pesticide in the study area, as it was also detected in the soil samples analysed. Lindane is in the WHO category II insecticide group and is capable of causing acute as well as chronic intoxication (Sosan et al. 2008). Although, the levels of lindane detected in the water samples did not exceed the WHO limit, exposure to large amount of lindane have been reported to have negative effects on the nervous system with symptoms from headaches and dizziness to convulsions and more rarely, death (Leena et al. 2012). The mean concentration of lindane recorded in this study was lower than the mean concentrations of 37.0 and 0.0578 µg/l reported by Idowu et al. (2013) and Olayinka (2013) in river water samples from cocoa producing areas in Ondo State Central Senatorial District, Nigeria and Ilawe-Ekiti, Ekiti State, Nigeria, respectively.

Alpha-endosulfan, an isomer of the parent chemical endosulfan, was detected in 26.3 % of the water samples analysed at an average of 0.03 ± 0.01 µg/l, which was higher than the WHO MRL of 0.01 µg/l for drinking water. In general, the mean concentration of α-endosulfan at distances 0–15 m was higher than the mean value recorded at distances 16–30 m. Distances above 30 m (control) recorded no alpha-endosulfan. However, with the exception of water samples from Nkrankwanta which recorded a mean alpha-endosulfan concentration of 0.01 µg/l at a distance 16–30 m, all the other sites with detectable residues recorded mean concentrations that were above the WHO MRL of 0.01 µg/l for drinking water. The mean residue concentration of α-endosulfan recorded in this study was lower than the mean values of 3200 and 0.597 µg/l recorded in river water samples from cocoa producing areas in Nigeria by Idowu et al. (2013) and Okoya et al. (2013a), respectively. Additionally, endosulfan-sulfate, a metabolite of endosulfan, was detected only in water samples from Nkrankwanta (0.02 µg/l) and Diabaa (0.04 µg/l) at distances 0–15 m and were all above the WHO MRL of 0.01 µg/l for endosulfan-sulfate in drinking water.

The occurrence of endosulfan in the water samples most likely reflects the current use of the pesticide. Considering that the use of endosulfan was officially discontinued only recently in Ghana (in 2009) (Hogarh et al. 2014), farmers may still have possession or access to old stock of this product (Obiri-Danso et al. 2011; NPASP 2012). On the other hand, the relatively low occurrence/detection of endosulfan-sulfate compared to alpha-endosulfan suggests that historical input of endosulfan has not metabolized to endosulfan-sulphate or there is a minimal input of endosulfan at present. Endosulfan is known to be easily absorbed by the stomach, the lungs and the skin (Sosan et al. 2008). Long-term exposure to low doses of endosulfan has been shown to cause endocrine disruptions, reproductive and developmental damage in animals and humans (Sosan et al. 2008; Leena et al. 2012). It can therefore be concluded that water pollution due to endosulfan poses a threat to consumers in the study area.

Dieldrin was only detected in water (at distances 0–15 m) from Nkrankwanta (<0.01 µg/l) and Krakrom (0.03 µg/l). The mean concentration was 0.03 µg/l, which was comparable to the WHO MRL of 0.03 µg/l for drinking water. The presence of dieldrin in the water samples suggests the degradation of aldrin to dieldrin in the environment as aldrin was not detected. However, current or direct application and bioaccumulation of dieldrin as an insecticide cannot be entirely ignored. The occurrence of dieldrin in the water samples from the various farms may be linked to its detection in the soils of the various farms from which water samples were taken.

*p,p′*-DDT, an isomer of DDT, was detected in 31.6 % of the water samples analysed at a mean concentration of 0.04 ± 0.00 µg/l. The mean *p,p′*-DDT concentration recorded at distances 16–30 m was comparatively higher than the mean value recorded at distances 0–15 m. Water samples taken at distances above 30 m (control) from cocoa farms recorded no *p,p′*-DDT concentration. The concentrations of *p,p′-*DDT recorded in this study were however below the WHO MRL of 2.00 µg/l for drinking water. The presence *p,p′*-DDT in the water samples confirmed the assertions made early, that there is a likely current application of pesticides which might contain DDT by farmers in the study area (probably with different trade names). It might also be as a result of previous contamination, as they degrade slowly and persist in the environment for a long time. Long-term exposure to low doses of DDT has been shown to affect the endocrine, reproductive systems, immune system and cause cancers (Afful et al. 2010; Okoya et al. 2013b). The mean concentration of *p,p′*-DDT recorded in this study was higher than the mean values of 0.03 and 0.02 µg/l recorded in river water samples from cocoa producing areas in Ilawe-Ekiti, Ekiti State, Nigeria (Olayinka 2013) and in Ondo State, Nigeria (Okoya et al. 2013a), respectively.

Generally, heptachlor was the most dominant pesticide residue recorded, occurring in 52.6 % of the water samples analysed at a mean concentration of 0.02 µg/l. The mean concentration recorded at the control site was generally low compared to those at distances 0–15 and 16–30 m. The mean concentrations of heptachlor recorded at the various sampling sites within the various distances were below the WHO guideline limit of 0.03 µg/l for drinking water except Kwakuanya (S4) at a distance 0–15 m and Krakrom (S3) at a distance 16–30 m which recorded mean concentrations of 0.03 and 0.04 µg/l which were comparable to and above the WHO MRLs of 0.03 µg/l, respectively. The high occurrence of heptachlor in the water samples suggests the continual usage of the illegal pesticide by cocoa farmers in the study area. It may also be attributed to previous contamination as well as environmental persistence from past usage of the chemical. Heptachlor is known to have implications in a broad range of adverse human health effects including endocrine disrupting properties, reproductive failures and birth defects, immune system malfunction, Parkinson’s disease and cancers (Afful et al. 2010; Okoya et al. 2013b), hence their presence is of serious concern. The mean heptachlor recorded in this study was higher than the mean value of 0.01 µg/l recorded in river water samples from cocoa producing areas in Ilawe-Ekiti, Ekiti State, Nigeria (Olayinka 2013).

### Relationship between soil physicochemical properties and pesticide residues detected

The relationship between soil properties and pesticide residues detected in the soil were analysed using the correlation of Pearson (Table 4). The strong positive correlation between soil pH and (lindane, dieldrin and beta-HCH) and a strong negative correlation with *p,p′*-DDT indicates that soil pH could have enhanced the adsorption and desorption of the pesticides, respectively. Soil pH influences the fate of chemical compounds in soil. Thus, an increase in soil pH resulted in a corresponding increase in the concentrations of lindane, dieldrin and beta-HCH while an increase in soil pH resulted in a decrease in levels of *p,p′*-DDT or vice versa. This finding is similar to an earlier work by Bentum et al. (2006) which reported significant negative correlations between extracted lindane and propoxur residues with soil pH, but contrary to another study by Aiyesanmi and Idowu (2012), which reported no significant (*p* > 0.05) correlations between soil pH and total organochlorine concentrations measured in soil samples from cocoa farms in Ondo State Central District in Nigeria.

**Table 4:** Values of Pearson’s correlation between soil physicochemical parameters and pesticide residues detected

Pesticides	pH	EC	%OC	%OM	%TN	NH_4_^+^	NO_3_^−^	Ava. K	Ava. P	Ex-K	%Sand	%Clay	%Silt
Lindane	0.753*	−0.492*	0.886**	−0.882*	−0.997**	0.645	0.204	−0.774*	0.864**	0.932**	−0.425	−0.970**	−0.863
Dieldrin	0.793*	0.172	0.998**	0.998**	−0.482	0.222	0.708*	−0.388	0.348	0.851*	−0.327	−0.951*	0.965**
HCH	0.959**	−0.167	0.932**	0.936**	−0.622	0.554	0.666	−0.168	0.308	0.925**	−0.997**	−0.943*	0.980**
DDT	−0.755**	0.034	−0.660*	−0.667*	0.141	−0.420	−0.886*	−0.402	0.297	−0.544	−0.637*	0.452	−0.700*

* Correlation is significant at the 0.05 level (2-tailed) ** Correlation is significant at the 0.01 level (2-tailed) *OC* organic carbon, *OM* organic matter, %TN percentage nitrogen, *Ava*-*P* available phosphorus, *Ava*-*K* available potassium, *EC* electrical conductivity, *Ex*-*K* exchangeable potassium, *NO*_*3*_ Nitrate, *NH*_*4*_^+^ ammonium, *HCH* beta-HCH, *DDT* *p,p′*-DDT

In addition, the strong positive correlation between organic carbon and (lindane, dieldrin, and beta-HCH) and a high negative correlation with *p,p′*-DDT indicates that an increase in soil organic carbon resulted in increased concentrations of lindane, dieldrin and beta-HCH while an increase in soil organic carbon resulted in decreased levels of *p,p′*-DDT or vice versa. The finding of this study is in line with a study which reported that both extracted lindane and propoxur residues of cocoa growing soils correlated negatively with organic carbon (Bentum et al. 2006).

Similarly, a strong positive correlation was observed between organic matter and (lindane, dieldrin and beta-HCH) which indicated that the pesticide residue levels in the soils are associated with high organic matter content of the soil and could be attributed to pesticide molecules having high tendency of binding to organic matter in soil, similar to fats or lipids of plants and animals (Swackhamer and Hites 1988; Bentzen et al. 2008). On the other hand, the negative correlation between organic matter and *p,p′*-DDT indicates that an increase in organic matter of the soils resulted in a corresponding decrease in *p,p′*-DDT and vice versa. This findings are similar to a study by Aiyesanmi and Idowu (2012) which reported a significant (*p* < 0.05) correlations between organic matter and total organochlorine pesticides measured in soil samples from selected cocoa farms.

Behaviour of pesticides in the soil environment is also greatly influenced by the soil texture. Sandy soils are known to facilitate leaching whereas the clayey soils help accumulation through colloid formation. The high negative correlation between percentage sand of soil and (*p,p′*-DDT and beta-HCH) indicates the influence of sand on extractable pesticides in soils. Thus, an increase in percentage sand of soils resulted in a corresponding decrease in *p,p′*-DDT and beta-HCH and vice versa. Similar studies by Bentum et al. (2006) and Aiyesanmi and Idowu (2012) reported no significant (*p* > 0.05) correlations between percentage of sand and extractable lindane and propoxur residues, and total organochlorine pesticides measured in soil samples, respectively.

In addition, the negative correlation between percentage clay of soil and (lindane, dieldrin and beta-HCH) suggests that percent clay has significant influence on the distribution of pesticides in soils. Thus, an increase in percentage clay of soils resulted in a corresponding decrease in lindane, dieldrin and beta-HCH and vice versa. This finding is contrary to a study by Aiyesanmi and Idowu (2012) which reported no significant (*p* > 0.05) correlations between percentage clay and total organochlorine pesticides measured in soil samples from selected cocoa farms. The high positive correlation between percentage silt and (dieldrin, lindane and beta-HCH) and a negative correlation between percentage silt and (*p,p′*-DDT) indicates that an increase in percentage silt resulted in increased concentrations of lindane, dieldrin and beta-HCH while an increase in percentage silt resulted in decreased levels of *p,p′*-DDT or vice versa. This finding however disagree’s with a study by Aiyesanmi and Idowu (2012) which reported no significant (*p* > 0.05) correlations between percentage silt and total organochlorine pesticides measured in soil samples from selected cocoa farms in Nigeria.

The positive correlation between nitrate and (dieldrin and beta-HCH), available phosphorus and (lindane), ammonium and (lindane and beta-HCH) and exchangeable potassium and (dieldrin, lindane and beta-HCH) indicates that these soil nutrients have significant influence on the distribution of pesticides in soils, hence an increase in levels of these nutrients resulted in a corresponding increase in their respective associated pesticide residues. Similarly, the negative correlation between percentage nitrogen and (lindane and beta-HCH), nitrate and (*p,p′*-DDT), available potassium and (lindane) and exchangeable potassium and (*p,p′*-DDT) indicates that an increase in the levels of these soil nutrients resulted in a decrease in their respective associated pesticide residue levels in the soils and vice versa.

## Conclusions

The results from this study show that residues of organochlorine pesticide are present in soil and water samples within and around cocoa farms in the Dormaa West District of Ghana. Seven banned organochlorine pesticides (dieldrin, lindane, beta-HCH, *p,p′*-DDT, alpha-endosulfan, endosulfan-sulfate and heptachlor) were detected. The occurrence of organochlorine pesticide residues in the samples may be attributed to the illegal use of the pesticides by farmers in the study area or due to their historic use, since organochlorine pesticides are prohibited from agricultural use in Ghana.

The concentrations of organochlorine pesticide residues recorded in the soil samples analysed were generally below their respective US MRLs for agricultural soils except for lindane at Kwakuanya (S4) and beta-HCH at Krakrom (S4) and Kwakuanya (S4) which were above the safe limits for agricultural soils. The organochlorine pesticide residues might have found their way into the soils via spray drift during spring of cocoa trees, wash-off from sprayed cocoa trees, accidental spillage, wrong disposal of left over spray solution, sprayer wash water and pesticide containers as well as misuse or overuse of the pesticide, among others. The presence of organochlorine residues in the soils may pose potential hazards to soil organisms and can pollute the surrounding water bodies through surface runoff and leaching. Moreover, there is the possibility of translocation of these residues from the polluted soils into cocoa beans through the root system, and also into other crops like vegetables that are commonly intercropped in cocoa farms.

The trends of organochlorine pesticide residues in the water samples analysed decreased with increase in distance away from cocoa farms (0–15 m > 16–30 m > above 30 m). The organochlorine pesticide residues recorded in water samples from the various sites (within the various distance categories to cocoa farms) were below their respective WHO MRLs for drinking water except for alpha-endosulfan at Diabaa (S2) and Kwakuanya (S4) at distance 0–15 m and at Kwakuanya (S4) at distance 16–30 m, endosulfan-sulfate at Nkrankwanta (S1) and Diabaa (S2) at distance 0–15 m and heptachlor at Krakrom (S3) at distance 16–30 m. The presence of organochlorine pesticide residues in the water samples could be due to drift during pesticide application, direct overspray, direct spillage, pesticides misuse, improper disposal of left over spray solution, sprayer wash water and pesticide containers, runoff from treated areas or leaching, among others. The concentrations measured however suggest that pesticide residues in hand-dug wells within the various distances do not pose health threats to cocoa farmers’ households except for wells from S1, S2, and S4 at distances 0–15 m and S3 and S4 at distances 16–30 m which recorded concentrations of some residues above their respective WHO MRL for drinking water.

Routine monitoring of pesticide residues in the study area is necessary for the prevention, control and reduction of environmental pollution, so as to minimize health risks to human. In addition, hand dug wells for domestic use should be sited above 30 m from cocoa farms to reduce potential pesticide contamination. The need to sensitize farmers on safe pesticide use is thus crucial to reduce the levels of pesticide residues in soils and drinking water in the study area.
